# The rationale for nephron-sparing surgery in unilateral non-syndromic Wilms tumour

**DOI:** 10.1007/s00467-023-06099-2

**Published:** 2023-08-21

**Authors:** Kiarash Taghavi, Sabine Sarnacki, Thomas Blanc, Olivia Boyer, Yves Heloury

**Affiliations:** 1 Department of Paediatric Urology, Monash Children’s Hospital, Victoria Melbourne, Australia; 2 Department of Paediatrics, Monash University, Victoria Melbourne, Australia; 3 Department of Pediatric Surgery, Urology and Transplantation, Hôpital Universitaire Necker-Enfants Malades, APHP, Université de Paris Cité, Paris, France; 4 Department of Pediatric Nephrology, MARHEA Reference Center, Imagine Institute, Hôpital Universitaire Necker-Enfants Malades, APHP, Université de Paris Cité, Paris, France

**Keywords:** Nephron-sparing surgery, Nephrectomy, Wilms tumour, Nephroblastoma, Kidney function, Outcome

## Abstract

The central question of nephron-sparing surgery in unilateral non-syndromic Wilms tumour sits at a crossroads between surgery, oncology, and nephrology. There has been a significant paradigm shift in paediatric oncology towards reducing toxicity and addressing long-term treatment-related sequalae amongst childhood cancer survivors. After paediatric nephrectomy and 30–50 years of follow-up, 40% of patients will have chronic kidney disease, including 22% with hypertension and 23% with albuminuria. It is difficult to predict which patients will progress to develop hypertension, reduced glomerular filtration rate, albuminuria, and a higher cardiovascular risk. For these reasons, nephron-sparing surgery when it is technically feasible must be considered. To decrease the incidence of positive surgical margins (viable tumour present at a resection margin), incomplete lymph node sampling, and complications, these procedures should be performed at specialist and experienced reference centres. Based on the impacts of individual treatment pathways, survivors of childhood WT need to be followed through adulthood for early detection of chronic kidney disease, hypertension, and prevention of cardiovascular events.

## Introduction

Survivors of childhood cancer remain at risk of reduced life expectancy and morbidity due to treatment-related effects [1]. In 2016, The Renal Tumour Study Group of the International Society of Paediatric Oncology developed a protocol that established new pathways in diagnosis and treatment of children with renal tumours, to continue the iterative process of collaboration and research. This was termed the UMBRELLA protocol and one of its goals was to decrease acute toxicity and long-term treatment-related sequelae [2]. The chosen surgical strategy plays an important role in preserving functional kidney and reducing morbidity through the selective utilization of nephron-sparing surgery (NSS) [3]. To preserve kidney function, NSS was established in children with bilateral Wilms tumours (WT), or unilateral WT with a syndrome predisposing to metachronous Wilms tumours. However, in the recent UMBRELLA protocol, NSS has become an acceptable treatment option in non-syndromic unilateral WT when certain criteria are met [2]. Adoption of NSS has been slow [3] due to concern around surgical complications, and positive surgical margins necessitating additional therapy [4].

The principles that have established the application of NSS in children include a physiological rationale for its use, consistent case selection, maintenance of the oncological principles of surgery, and surgery that is performed in a high-volume centre by an experienced team. These themes will subsequently be discussed in detail. The goal of this review was to determine a rationale for the adoption of NSS in a wider context and to specify the conditions, extended indications, technical considerations, and expected outcomes.

## Rationale for a broader application of nephron-sparing surgery

The goal of NSS is to safely preserve as much functioning and draining renal parenchyma as possible while maintaining an excellent oncological outcome. To determine the rationale for this, the consequences of total nephrectomy (TN) on kidney function, and the associated cardiovascular morbidity, must be established. The evolution of kidney function with age and the consequences of TN in children or adults can be illustrated by studying various situations. The definition of kidney injury and classification of chronic kidney disease are based on the glomerular filtration rate (GFR) and albuminuria, as standardized by the KDIGO CKD work group [5, 6]. Furthermore, when eGFR is reported, it should be accompanied by the endogenous filtration marker, assay method, and estimating equation. In this consensus statement, criteria and severity for albuminuria and proteinuria are also precisely defined.

## The physiology of kidney function and the aging kidney

GFR is low at birth and reaches adult levels at the end of the second year of life [7]. It is stable at around 120 ml/min/1.73 m^2^ until the fourth decade, after which time it slowly declines by 8 ml/min/1.73 m^2^ per decade [8]. This decline relates to reduced renal mass, glomerular sclerosis, and reduced glomerular capillary flow. There is wide anatomical variation in the total glomerular number, between as little as 200,0000 and nearly 2 million [9, 10]. The number of nephrons present at birth relates to the fetal milieu and genetic factors. Of the many aspects involved, birth weight has been the most widely studied and there is a linear relationship between nephrons at birth and birth weight (260,000 nephrons per kg) [11]. However, it is noted that there is a huge variation around the line of association. Interestingly, the importance of birth weight holds for pre-term infants with appropriate weight for age, or term infants who are small for gestational age [12].

The decline of GFR with age is often undetected because of concomitant decrease in muscular mass [13], notwithstanding that creatinine is not a pure marker of glomerular filtration (it is secreted and reabsorbed by kidney tubules) [14]. Cystatin C is produced by all nucleated cells, is freely filtered by glomeruli and not reabsorbed, and the filtration rate appears unaffected by severe illness [15]. Ultimately, when an accurate GFR assessment is needed a measured GFR should be used [16, 17].

Reduction of GFR with age is of growing importance as life expectancy in developed countries has increased significantly, and kidney failure carries a significant health burden. The diminishing function of the aging kidney is also affected by common co-morbidities (hypertension, nephrotoxic medications, diabetes, and metabolic syndrome). For these reasons, the mean eGFR in the general population over 70 years of age is less than 60 ml/min/1.73 m^2^ [13]. A reduction of GFR (< 45 ml/min/1.73 m^2^) in adults over 60 years of age doubles all-cause mortality and also has a significant impact on cardiovascular mortality [18]. This notion of the aging kidney is critical when we interpret the consequences of TN in paediatric patients. It underscores that even 20–30 years of follow-up is insufficient to determine the consequences of total nephrectomy.

## Single kidney (congenital or acquired) in children

In 1981, Brenner postulated that hyperfiltration in remnant nephrons following renal ablation contributes to progressive kidney dysfunction after studying rats [19]. Decreased functional kidney reserve leads to increased intraglomerular pressure and hyperfiltration. These physiological alterations may exacerbate any co-existent predisposition to glomerular pathology, whether genetic or acquired. Judicious and pro-active management of risk factors in patients with acquired single kidney (ASK) may slow the progression to chronic kidney disease [14].

Congenital and acquired causes of single kidney have distinct consequences. Nephrogenesis continues up to 36 weeks’ gestation and this allows an opportunity for physiological compensation in situations where there is a congenital single functioning kidney [20]. This phenomenon could explain why cases of congenital single kidney (CSK) may have a superior functional profile, although this has been variably found in comparative studies (Table 1). However, children with both CSK and ASK do show compensatory hypertrophy regardless of aetiology [21], and it may be this adaptive (or maladaptive) response to reduced kidney reserve is more age and stage dependent rather than related to aetiology per se. Furthermore, a higher GFR within the first decade in children with a single kidney needs to be interpreted with caution, as this may represent a higher degree of hyperfiltration and herald a worst prognosis, rather than reflect better kidney function.

**Table 1:** Summary of studies comparing outcomes of congenital and acquired solitary kidney

Country (year)	Population	Age (years)	Mean follow-up (years)	Outcome (CSK vs. ASK)	*p*-value
France (2011) [22]	*n* = 97 (CSK 44, ASK 53)	2–25 years	9	GFR (107 vs. 95)	*p* < 0.01
Korea (2019) [23]	*n* = 217 (CSK 153, ASK 64)	N/A	5 (mean per participant of entire study)	Hazard ratio for CKD comparing to normal subjects CSK: 6.2 (95%CI 2.3–16.5) ASK: 2.2 (95%CI 0.8–5.9)	
France (2021) [21]	*n* = 210 (CSK 143, ASK 67)	13		Hazard ratio for GFR: 0.06 (95%CI: − 0.08 to 0.2)	*p* = 0.4
Turkey (2022) [24]	*n* = 204 (CSK 75, ASK 129)	57 (mean)	6.5	eGFR at last admission (69 vs. 58) No difference in annual decline in eGFR	*p* = 0.01 *p* = 0.720
Netherlands (2023) [25]	*n* = 818 (CSK 715, ASK 103)	Paediatric range (not specified)	13 (median)	Severe kidney injury at 18 years of age (39% vs. 37%)	
Turkey (2023) [26]	*n* = 101 (CSK 71, ASK 30)	13 (mean)	9	eGFR (120 vs. 110)	*p* < 0.001

A review of adults following childhood TN (for oncological and non-oncological indications) revealed that after > 30 years follow-up: 40% had kidney dysfunction (eGFR < 90 ml/min/1.73 m^2^), 22% had hypertension, and 23% had albuminuria. After 50 years of follow-up 88% of patients had an eGFR between 46 and 86 ml/min/1.73 m^2^ [27]. These results must be put in the context of a heterogenous group, with 30 years of follow-up and historical regimens of both adjuvant chemotherapy and radiotherapy. Nonetheless, nephrectomy performed for non-oncological indications had similarly poor outcomes (kidney dysfunction 38%, hypertension 26%, albuminuria 33%) after >30 years of follow-up. Care pathways and recommended follow-up have been developed for this population in response to the risks outlined [20, 22, 27, 28].

## Living kidney donors

Although the effects of nephrectomy in childhood and adulthood are quite distinct from an experimental perspective [29], living kidney donors represent a valuable model to understand the evolution of kidney function after TN, being generally healthy and well-studied adults. However, it must be noted that living kidney donors have a higher incidence of developing metabolic syndrome than matched controls [30]. This may partially explain their long-term cardiovascular morbidity, but GFR has been identified as an independent cardiovascular risk factor in this group [31]. Another major limitation of extrapolating outcomes from this group, over and above the differences between donor nephrectomy and ASK in childhood, are the requirement for at least 10 years of follow-up to allow some meaningful application.

A study of nearly 4000 living donors with a mean follow-up of 17 years illustrated some of the expected dysfunction in ASK. Proteinuria was detected in 6%, eGFR < 60 ml/min/1.73 m^2^ in 36%, and 2.5% had an eGFR < 30 ml/min1.73 m^2^ or kidney failure (KF). The cumulative incidence of kidney failure was 1.3 per 1000 donors at 15 years, 7.7 per 1000 donors at 30 years, and 8.8 per 1000 donors at 40 years [32]. These figures must be interpreted with some care. The risk of kidney failure is elevated in related donors, which may be due to ill-defined genetic factors, or more comprehensive and long-term study of related donors (unrelated donation was rare before the 1980s) [32, 33].

Another North American study of over 70,000 kidney donors reported the cumulative incidence of kidney failure at 15-years post-donation. This ranged from 1.1 per 1000 donors to 3.3 per 1000 donors. This was related to eGFR six months post-donation, but importantly the pre-donation eGFR was not predictive of post-donation kidney function [34].

## Specific factors affecting kidney function in cases of Wilms tumour

Treatment effects from chemotherapy and radiotherapy on kidney function are an important additional consideration in this population. A cohort of children (*n* = 75) treated for non-syndromic WT with TN alone (not requiring nephrotoxic chemotherapy or radiotherapy) have illuminated this point. With a mean follow-up of 20 years, 21% had an eGFR of < 90 ml/min/1.73 m^2^; and no patient had an eGFR of < 60 ml/min/1.73 m^2^. The authors concluded in this specific population the risk of developing significant long-term kidney dysfunction was low [35]. However, the consequences of mild kidney impairment in later adulthood remain unclear.

A smaller (*n* = 37) but prospective German study had a longer mean follow-up of 25 years. Of note, in this population of children treated for WT, 60% received radiotherapy (predominantly flank/abdominal), and 14% nephrotoxic chemotherapy (cyclophosphamide and/or carboplatin). They found 27% of patients had an eGFR between 60 and 90 ml/min/1.73 m^2^, and 3% had an GFR < 60 ml/min/1.73 m^2^. Hypertension was present in 41% of patients. The results when compared with previous studies (in terms of kidney function and hypertension) are likely explained by the extended follow-up and impact of adjuvant treatment [36].

A Dutch study recently reported the outcomes of 31 WT survivors with 15 years of follow-up. The rates of hypertension were 26% and 4% of patients had an eGFR < 60 ml/min/1.73 m^2^ [37]. And even more recently a French childhood survivor cancer study reported outcomes of 5498 survivors and 42,118 person-years of follow-up. They reported that children who underwent nephrectomy and required ifosfamide (> 60 g/m^2^) had an extremely high risk of hospitalisation for renal causes [38]. From these studies, there is a proportion of patients treated for WT following TN who will have chronic kidney disease and/or hypertension. However, the relative renal impact of surgery, radiotherapy, and nephrotoxic chemotherapy needs to be more clearly delineated [16]. Furthermore, until *WT1* genetic screening is uniformly applied, it is difficult to identify if there is a sub-group of children with poor outcomes who are incorrectly classified as non-syndromic [39].

A large prospective childhood cancer survivor study reported kidney outcomes in children with unilateral non-syndromic WT stratified by treatments received (*n* = 2008) [40]. Compared to their siblings, WT survivors experienced a ten-fold increased in the rate of kidney failure (2.4% 35-year cumulative incidence) [40]. The addition of abdominal radiotherapy doubled the relative risk of kidney failure to 20-fold [40]. Although this was a North American study, there was no survivor group that underwent nephrectomy *alone*. Once long-term follow-up for this group becomes available for comparison, it will further clarify the consequences of adjuvant treatments on long-term outcomes.

**Fig. 1:**
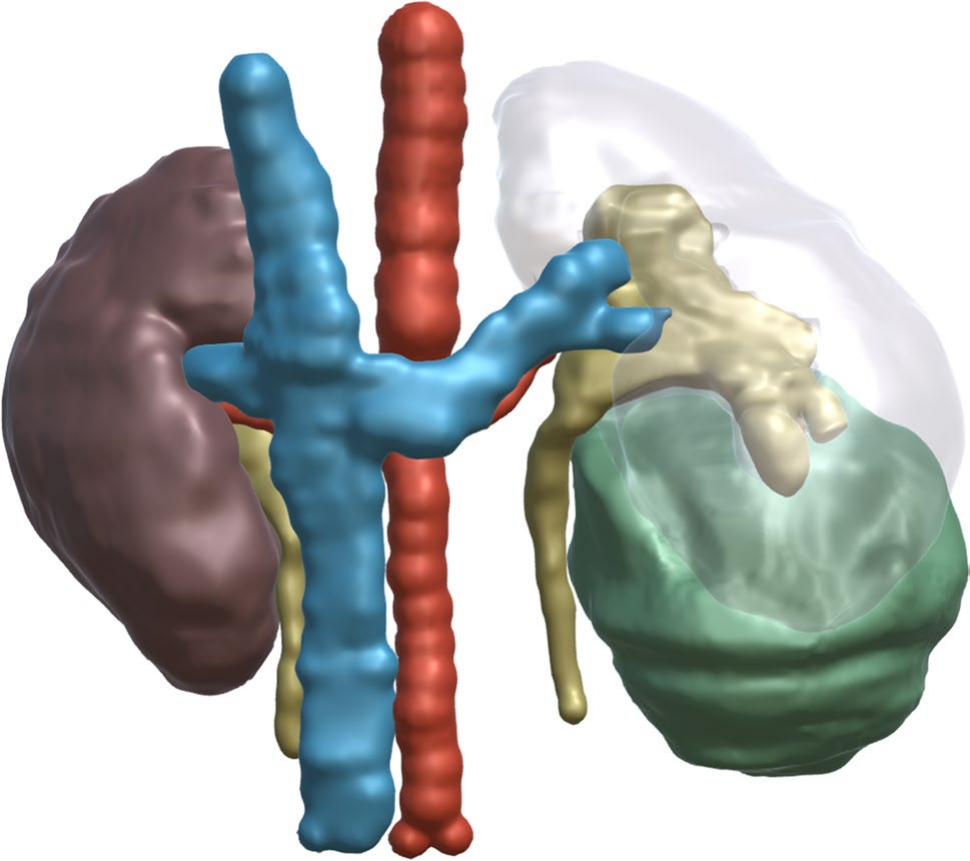
Anterior view of a 3D reconstruction illustrating a lower pole Wilms tumour (green) abutting the inferior calyx but well clear of the left renal vein and artery

In the UMBRELLA protocol, post-operative local or flank radiotherapy is indicated in children with unilateral WT with:

1. Intermediate-risk histology and stage III disease (lymph node positivity, R1 or R2 resection, e.g. microscopic or macroscopic residual disease, or tumour rupture)
2. High-risk histology stage II disease (except blastemal subtypes)
3. High-risk histology stage III disease (all histological subtypes)

**Fig. 2:**
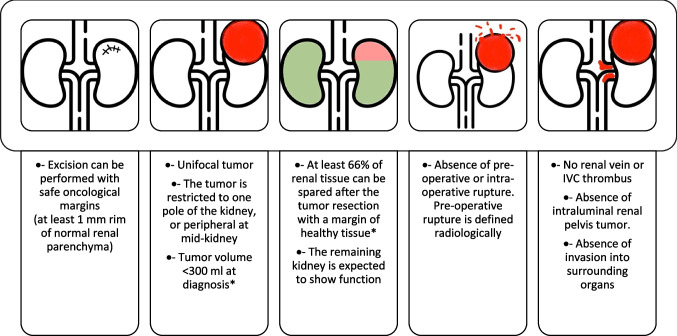
Conditions for NSS in unilateral non-syndromic WT from the UMBRELLA protocol (2016) established by the Renal Tumour Study Group of the International Society of Paediatric Oncology. The points marked “*” are discussed further in the manuscript

It must be emphasised that many aspects of this decision can only be confirmed post-operatively following histological review (e.g. histological risk group, lymph node positivity, tumour at resection margins). Dosing depends on many individual and disease factors, but the protocol stipulates that the dose to the whole kidney should not exceed 12 Gy when NSS is performed, even if there is high-risk histology. The anticipated use of radiotherapy is used as an argument against NSS when there is a high suspicion of lymph node involvement because the effects of radiotherapy are said to counterbalance any benefit of nephron capital linked to NSS (extension to adjacent organs being a contra-indication of NSS per se). However, radiotherapy-associated kidney injury is a very slow process (particularly in association with the protocolised reduction in radiotherapy dose) and is imprecisely defined (but includes hypertension [41, 42] and functional effects). Therefore, although circumstances that may mandate radiotherapy should be considered in decision-making around performing NSS, a rationale for it remains so long as the oncological principles of surgery can be maintained (further discussed under “Nephron-sparing surgery” and illustrated in Fig. 2).

### Cardiovascular morbidity and mortality

Long-term studies of WT survivors demonstrate significant cardiovascular morbidity and mortality into adulthood [43]. A recent study from the Childhood Cancer Survivor Study (of which 7–9% had WT) confirmed that life expectancy has improved amongst adult survivors of childhood cancer. The gap for life expectancy (compared with individuals without a history of childhood cancer) was 25% for those diagnosed in 1970–1979, 19% for those diagnosed in 1980–1989, and 14% for those diagnosed in 1990–1999 [1].

The risk of adverse health outcomes was well defined by a large cohort of WT survivors (*n* = 1441) in the British Cancer Survivor Study [43]. Cumulative risk of death increased from 5.4% at 30 years from diagnosis, to 22.7% at 50 years from diagnosis. Three-quarters of excess deaths beyond 30 years were attributable to second primary malignancies (50%) and cardiac diseases (25%). Radiotherapy exposure was a risk factor for both outcomes [43].

The same group confirmed that survivors of childhood cancer experienced three times the number of cardiac deaths compared to the general population. Of all the primary tumour types included, WT survivors were found to have the greatest risk of ischemic heart disease. This was attributed to hypertension and having a solitary kidney, as cardiac radiotherapy exposure in these survivors would be anticipated to be less than the comparator tumour groups (e.g. Hodgkin lymphoma) [44]. Childhood cancer survivors are not immune to the host of cardiovascular risk factors that plague the general population: obesity, diabetes mellitus, smoking, positive family history of myocardial infarction/stroke, arterial hypertension, and dyslipidaemia; in fact they experience higher rates of the latter two [45]. In children surviving renal tumours, heart failure in particular leads to a 15-fold increase in hospitalisation compared to the general population [46]. Although this should be interpreted in the context of historical cohorts, nonetheless these studies illustrate the importance of minimising the long-term cardiovascular impacts of treatment in children with renal tumours.

## Nephron-sparing surgery

### Conditions for NSS

Three conditions are necessary to develop NSS.

- Expertise: As NSS will represent no more than 15% of WT (including bilateral and syndromic WT), it is imperative to identify a few reference centres to concentrate expertise in each country (depending on population size). In adult urology, outcomes of robot-assisted partial nephrectomy closely relate to centre volume [47]. Low-volume centres were defined as those that performed less than 20 cases per year, and high volume 45–70 cases per year. Positive surgical margins (26% vs. 17%), length of stay (6 days vs. 4.7 days), warm ischaemia time (20 min vs. 15 min), operative time 181 min vs. 150 min), and major complications (12% vs. 4%) all improved significantly with higher centre volume [47]. In paediatrics, such cases are discussed at a regional multidisciplinary meeting at diagnosis. Definitive evaluation is performed at the reference centre following neoadjuvant chemotherapy. This expertise is necessary to maintain an excellent oncological outcome decreasing the risk of surgical up-staging and complications.
- Neo-adjuvant chemotherapy: The efficacy of neo-adjuvant chemotherapy is well documented in terms of reduction in tumour size and prevention of tumour rupture. Intra-operative tumour rupture may be reported by the surgeon or pathologist. It is defined by the presence of viable tumour cells at the surface of the specimen (beyond the tumour pseudocapsule). Primary nephrectomy carries a 9.7% rate of intra-operative tumour spill [48], versus 2.8% following neoadjuvant chemotherapy [49]. In a review of 75 patients with WT, NSS was feasible in 3% at diagnosis, which increased to 15% following neo-adjuvant chemotherapy [50].
- Imaging techniques: : A precise understanding of the relationship of the tumour with segmental renal vessels, major calyces, and the renal pelvis is critical to confirm the indication and determine the technique. Spatial understanding of these structures can now be appreciated with 3D reconstructions/printing based on CT scan images [51, 52] (Fig. 1). These provide a more intuitive anatomical assessment between the relationship of the tumour, arteries, veins, and collecting system, helping paediatric oncology surgeons in decision-making regarding NSS [52]. Furthermore, adult studies of partial nephrectomy confirm that inclusion of 3D virtual models: improves post-operative kidney function [53], and results in fewer collecting system injuries [54], and less transfusion requirement [54]. Intraoperative US can also be employed to visualize the critical relationships of the tumour and to aid in determining resection margins [55].

To allow accurate assessment of the impact of NSS, functional studies (DMSA scan) should be performed six months post-operatively. The UMBRELLA protocol also allows for a DMSA scan pre-operatively to clarify the anticipated contribution of spared renal tissue where this is equivocal.

### Indications

From a surgical perspective, the possibility of NSS should be at the forefront of the surgeons’ mind following neoadjuvant chemotherapy for WT. However, from the International Society of Paediatric Oncology WT trial 2001 (published in 2014), NSS was only employed in 3% of cases (whereas based on the literature one would have expected 5–10% of cases to be eligible for NSS) [3]. When reviewing participating Children’s Oncology Study group centres broadly (in the context of very low-risk and small tumours), only 8% of cases were deemed possible based on pre-treatment imaging [56]. There is an intrinsic challenge in developing treatment protocols that are to be applied in a variety of geographic locations and co-operative centres. The post-operative pathological specimen review suggests NSS may be applied in 25% of children with unilateral WT following neoadjuvant chemotherapy [57]; nonetheless, even large-volume single centres only report application as possible in 4–9% [35]. The reason for this discrepancy has not been clarified.

In the UMBRELLA protocol, conditions for NSS in unilateral non-syndromic WT were established (see Fig. 2). In a feasibility study of 75 unscreened unilateral non-syndromic WT, a blinded review of pre-operative CT scans utilizing the above criteria yielded 6% of patients were potentially amenable to NSS. The necessity to preserve greater than two-thirds of renal parenchyma is somewhat arbitrary, and details of how exactly this is to be calculated are not provided in the protocol. If this number is adjusted to 50%, NSS could be applied to 9% of patients, and if more than one-third of renal parenchyma is to be preserved 15% of patients may undergo NSS [50]. Although the ability to preserve functional renal parenchyma is an important consideration, we believe the relationship of the tumour to critical hilar structures is paramount.

In a small but detailed study of NSS in unilateral WT, an excellent functional recovery was observed in patients with > 40% preserved volume, with the preserved kidney demonstrating catch-up growth and function on DMSA scans during adolescence [58].

A further questionable criterion is initial tumour volume (< 300 mL) [2]. This is rationalized in the protocol by the fact that lymph node positivity is the exception rather than the rule in small tumours (5.5%) [59]. Both major study groups have confirmed that tumour volume correlates with lymph node positivity [60, 61]. A smaller tumour < 4 cm is also likely to be associated with a more benign eventual pathological diagnosis [62].

Lymph node positivity would necessitate radiotherapy as previously discussed, whose long-term impact on the remaining renal parenchyma remains ill-defined (further discussed under “Specific factors affecting kidney function in cases of Wilms tumour”). The effectiveness of lymph node sampling in the context of NSS has been questioned by a SEER (Surveillance, Epidemiology and End Results) database study. This study indicated that regional lymphadenectomy was omitted much more frequently when NSS was performed as opposed to TN (56% vs. 16%, *p* < 0.001) [63]. This is not because of technical limitations, but rather a surgical misconception or omission when performing NSS, and should be improved in the future.

### Technical aspects

In unilateral non-syndromic WT, partial nephrectomy with a rim of normal parenchyma has been advocated by surgeons, pathologists [61], and in protocols [2], in an effort to decrease the risk of incomplete resection. This is a concept that is useful to prevent positive surgical margins, although the recommended margin of normal parenchyma has not been clarified. However, there are some circumstances where this rule may be excessive. In a polar exophytic tumour where the rim of normal parenchyma is thin, following neoadjuvant chemotherapy a pseudocapsule usually forms. If there is no rupture of the lesion at this point, and the surgical margins are clear otherwise, NSS may be considered safe. Laparoscopic-assisted open procedures [64] and robotic-assisted laparoscopic [65] partial nephrectomy have both been described with success. It should be emphasised however that NSS utilising a transperitoneal minimally invasive approach is not recommended because of the risk of massive spillage if margins are not secured, made worse by insufflation, and necessitating whole abdominal irradiation.

Regardless of the approach and depending on tumour localization, it can be necessary to temporarily clamp the renal artery, which can be safely done for up to 30 min [66]. Other authors advocate for a “zero-ischemia” approach, avoiding any major vessel clamping [58, 64]. Even complex closure of the collecting system can be safely performed without utilizing a JJ stent [67], which has the benefit of not interfering with ureteric peristalsis [68]. Lymph node sampling is then performed in the hilar, ipsilateral paraaortic, and caval nodes [69], with the intention of obtaining at least seven lymph nodes [2].

### Complications

The largest and most comprehensive report comparing the complication profiles of NSS vs. TN suggested a higher rate of complications associated with NSS (36.4% vs. 13%). Prolonged urinary leak (persisting > 5 days following surgery) was the most common complication and the only complication with a statistically significant difference between groups (16% vs. 0%, *p* = 0.003) [67]. It must be noted that the majority of NSS in this study was done in the context of bilateral WT which is by nature more complex. When unilateral non-syndromic WT alone are compared, the difference in complication rate appears less pronounced (10% vs. 5%, *p* = 0.052) [3].

Nonetheless, surgical complications including urinary leak are problematic, requiring drainage that classically delays the resumption of adjuvant chemotherapy.

## Oncological outcomes

In the International Society of Paediatric Oncology WT trial 2001 (published in 2014) the overall survival and event-free survival in unilateral WT were compared for NSS (91 patients) and TN (2709 patients). Both overall survival (NSS: 100% vs. TN: 94.4%, *p* = 0.06) and event-free survival (NSS: 94.8% vs. TN: 86.5%, *p* = 0.06) were similar amongst the two groups. However, in the NSS group, the tumours were smaller and more often limited to the kidney (65% vs. 48%). In this study, the incidence of positive surgical margins in the NSS group was 9% (8/91) and the incidence of positive surgical margins in the TN was 13% (355/2709) [3]. In a separate study, relapse rates were also found to be identical between NSS and TN (when stage and grade of tumour are controlled for) [70].

When considering NSS, the incidence of positive margin is critical. Flank radiotherapy will have consequences on the residual renal parenchyma, although this has been difficult to define [41]. The long-term impact of radiotherapy has also been well documented, with reduced life expectancy [1], and higher rates of second primary malignancies [71].

## Kidney outcomes

Because of limited numbers and follow-up of patients with unilateral non-syndromic WT that have undergone NSS, broad and long-term evaluation of kidney function is not well documented. One long-term study following children after NSS or TN for renal tumours into the fifth decade showed significantly higher eGFR measurements associated with NSS, both after the second decade (110 ml/min/1.73 m^2^ vs. 91.2 ml/min/1.73 m^2^), and at last follow-up (110 ml/min/1.73 m^2^ vs. 95 ml/min/1.73 m^2^, *p* = 0.02) [72]. Although other studies with shorter follow-up are less impactful, a recent meta-analysis of all studies confirmed higher eGFR in children undergoing NSS (116 ml/min/1.73 m^2^ vs. 98 ml/min/1.73 m^2^) [73]. Recommendations for surveillance of kidney function and hypertension in WT survivors have been published, although they are based on personal opinion, and extrapolation from solitary kidney and cancer survivorship literature [74].

## Conclusion

In the long term, a proportion of patients undergoing TN for unilateral non-syndromic WT will experience a decline in kidney function, hypertension, and/or cardiovascular morbidity. These patients cannot be accurately predicted prior to surgery. For these reasons, NSS must be considered and discussed with patients in whom it is technically feasible, in an effort to reduce morbidity and mortality into adulthood. To decrease the incidence of positive surgical margins, incomplete lymph node sampling, and complications, these procedures should be performed at specialised and experienced reference centres. Based on the impacts of individual treatment pathways, survivors of childhood WT need to be followed throughout adulthood for nephroprotection and to monitor kidney function, blood pressure, and albuminuria, and prevent cardiovascular events.

## Abbreviations

NSS: Nephron-sparing surgery
WT: Wilms tumour
TN: Total nephrectomy
GFR: Glomerular filtration rate
CSK: Congenital single kidney
ASK: Acquired single kidney
